# Vitamin D and Immune Regulation: Antibacterial, Antiviral, Anti‐Inflammatory

**DOI:** 10.1002/jbm4.10405

**Published:** 2020-09-15

**Authors:** Emma L Bishop, Aiten Ismailova, Sarah Dimeloe, Martin Hewison, John H White

**Affiliations:** ^1^ Institute of Immunology and Immunotherapy University of Birmingham Birmingham UK; ^2^ Department of Physiology McGill University Montreal Quebec Canada; ^3^ Metabolism and Systems Research University of Birmingham Birmingham UK; ^4^ Department of Medicine McGill University Montreal Quebec Canada

**Keywords:** VITAMIN D, ANTIBACTERIAL, CATHELICIDIN, HEPCIDIN, β‐DEFENSIN 2, NOD2, AUTOPHAGY, INFLAMMATION, Th1, Th17, Treg

## Abstract

Regulation of immune function continues to be one of the most well‐recognized extraskeletal actions of vitamin D. This stemmed initially from the discovery that antigen presenting cells such as macrophages could actively metabolize precursor 25‐hydroxyvitamin D (25D) to active 1,25‐dihydroxyvitamin D (1,25D). Parallel observation that activated cells from the immune system expressed the intracellular vitamin D receptor (VDR) for 1,25D suggested a potential role for vitamin D as a localized endogenous modulator of immune function. Subsequent studies have expanded our understanding of how vitamin D exerts effects on both the innate and adaptive arms of the immune system. At an innate level, intracrine synthesis of 1,25D by macrophages and dendritic cells stimulates expression of antimicrobial proteins such as cathelicidin, as well as lowering intracellular iron concentrations via suppression of hepcidin. By potently enhancing autophagy, 1,25D may also play an important role in combatting intracellular pathogens such as *M. tuberculosis* and viral infections. Local synthesis of 1,25D by macrophages and dendritic cells also appears to play a pivotal role in mediating T‐cell responses to vitamin D, leading to suppression of inflammatory T helper (Th)1 and Th17 cells, and concomitant induction of immunotolerogenic T‐regulatory responses. The aim of this review is to provide an update on our current understanding of these prominent immune actions of vitamin D, as well as highlighting new, less well‐recognized immune effects of vitamin D. The review also aims to place this mechanistic basis for the link between vitamin D and immunity with studies in vivo that have explored a role for vitamin D supplementation as a strategy for improved immune health. This has gained prominence in recent months with the global coronavirus disease 2019 health crisis and highlights important new objectives for future studies of vitamin D and immune function. © 2020 The Authors. *JBMR Plus* published by Wiley Periodicals LLC on behalf of American Society for Bone and Mineral Research.

## Introduction

Vitamin D is obtained naturally from limited dietary components or from photochemical and thermal conversion of the cholesterol precursor 7‐dehydrocholesterol in skin in the presence of adequate ultraviolet B radiation. Cutaneous vitamin D synthesis occurs efficiently only when the angle of the sun is above 45 degrees. As a result, it is not produced in many major population centers of North America or Europe for 6 months of the year or more.^(^ ^1^ ^)^ To become biologically active, dietary or cutaneous vitamin D undergoes sequential hydroxylations, predominantly hepatic 25‐hydroxylation catalyzed by CYP2R1 and other enzymes,^(^ ^2^, ^3^, ^4^ ^)^ followed by CYP27B1‐catalyzed 1α‐hydroxylation in peripheral tissues.^(^ ^5^ ^)^ The major circulating metabolite of vitamin D, 25‐hydroxyvitamin D (25D) has a half‐life of several weeks and varies seasonally with fluctuations in cutaneous vitamin D synthesis. The active form of vitamin D, 1,25‐dihydroxyvitamin D (1,25D), binds to the vitamin D receptor (VDR), a member of the nuclear receptor family of ligand‐regulated transcription factors, and exerts its physiological effects largely, but not exclusively, through regulation of gene transcription.^(^ ^6^ ^)^ Notably, the most strongly induced 1,25D target gene encodes CYP24A1, the enzyme that initiates catabolism of both 25D and 1,25D, and thus acts as a physiological negative feedback loop.

The most recognized functions of the vitamin D metabolic and signaling system relate to its classical effects on musculoskeletal health.^(^ ^7^ ^)^ However, in recent years there has been an exponential increase in studies of nonclassical, extraskeletal actions of vitamin D.^(^ ^8^ ^)^ Prominent among these has been the growing body of evidence linking vitamin D with the immune system. The potential role of vitamin D as an endogenous regulator of both innate and adaptive immunity has garnered considerable attention in the last few months because of the apparent prevalence of vitamin D deficiency in communities where coronavirus disease 2019 (COVID‐19) infection and disease severity is equally pronounced. With this in mind, the aim of the current review is to provide a definitive overview of our current understanding of the immunoregulatory actions of vitamin D, with discussion in the final sections of how this may impact viral infection and the current COVID‐19 pandemic.

## Vitamin D Metabolism in the Innate Immune System

Vitamin D was discovered as the cure for nutritional rickets, and 1,25D was originally considered to be solely a calcium homeostatic hormone. However, there are links between vitamin D or sun exposure and infections that go back to the use of heliotherapy by the ancient Greeks to treat phthisis (tuberculosis [TB]).^(^ ^9^ ^)^ The concept re‐emerged in the mid‐1800s with the advent of the sanatorium movement in Europe to treat TB, and the subsequent demonstration that ultraviolet (UV) light could treat cutaneous TB (lupus vulgaris).^(^ ^10^, ^11^ ^)^ Recently, epidemiological observations have provided evidence for a protective role of vitamin D in autoimmune conditions (such as multiple sclerosis [MS] and type 1 diabetes mellitus [T1DM]) and infectious diseases (eg. respiratory tract infections).^(^ ^12^, ^13^, ^14^ ^)^ A rapidly expanding series of preclinical studies have shown that vitamin D signaling is active in both the innate and adaptive arms of the immune system.^(^ ^15^, ^16^ ^)^ The innate immune system is hardwired to detect an array of pathogens through numerous so‐called pattern recognition receptors (PRRs). Although there are a variety of PRRs, the two principle classes are the Toll‐like receptors (TLRs) and the NOD‐ (nucleotide‐binding oligomerization domain‐) like receptors. PRRs are activated by a variety of receptor‐specific pathogen‐associated molecular patterns (PAMPs).^(^ ^17^ ^)^ PRR signaling leads to the production of antimicrobial peptides, which have direct antibacterial and antiviral actions, as well as the production of numerous cytokines that recruit other components of the immune system to the site of infection.

**Fig 1:**
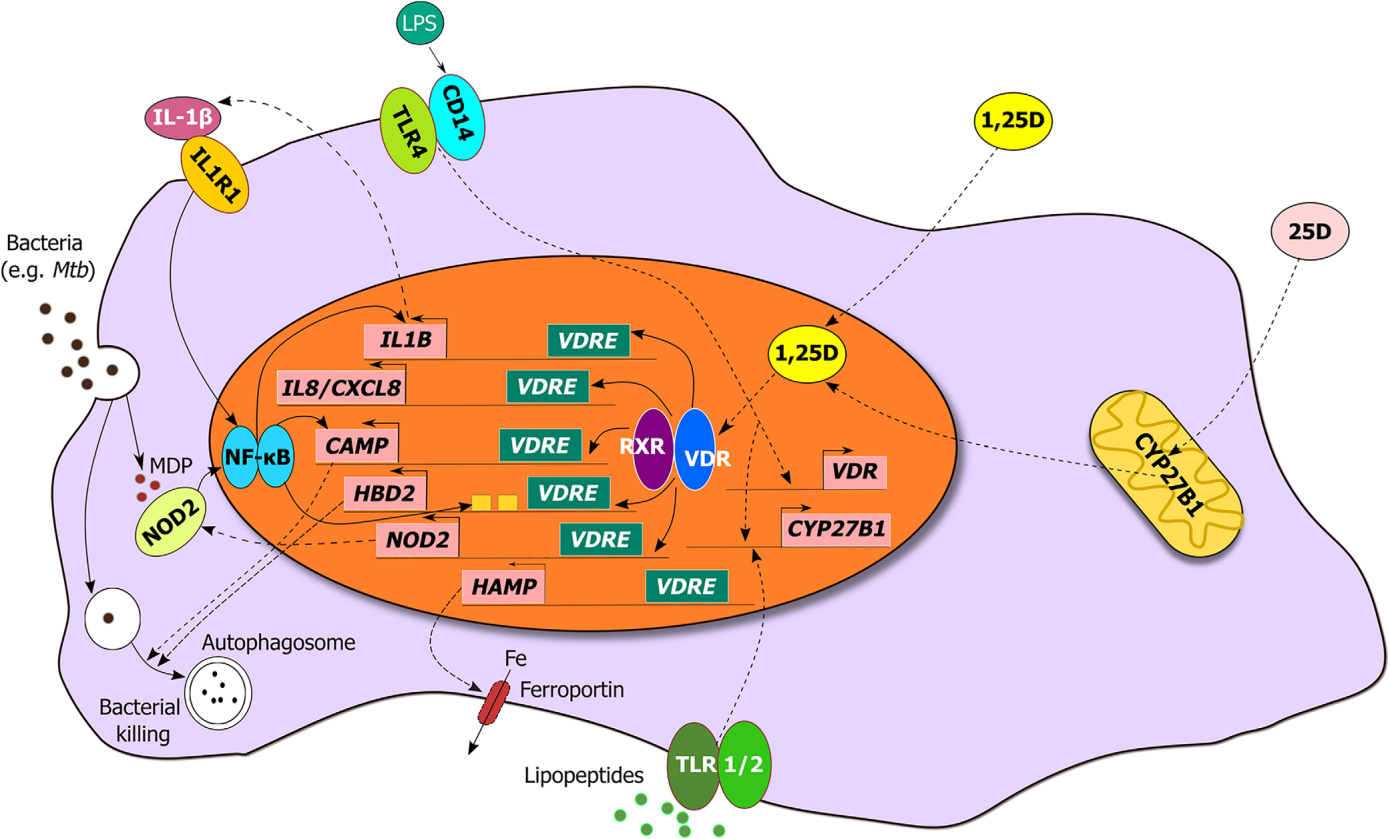
Vitamin D metabolism and innate immune signaling in the monocyte/macrophage. Schematic representation illustrating the regulation of expression of genes that encode antimicrobial peptides, pattern recognition receptors, and cytokines/chemokines by the 1,25D‐bound vitamin D receptor (VDR). In response to 1,25D, the VDR induces expression of the pattern recognition receptor NOD2, antimicrobial peptides CAMP and HBD2/DEFB4, and cytokines IL‐1β and IL‐8. The 1,25D‐VDR complex also functions to suppress *HAMP* expression, leading to increased ferroportin‐mediated export of iron. Upon stimulation with the ligand muramyl dipeptide (MDP) generated by the breakdown of bacterial peptidoglycan, NOD2 and IL‐1β can enhance *HBD2*, *CAMP*, and *IL1B* expression.

The paradigm shift in the perception of the physiological roles of vitamin D began with the finding that CYP27B1 is widely expressed in tissues that are unrelated to calcium homeostasis.^(^ ^5^, ^18^ ^)^ Similarly, the vitamin D receptor (VDR) is widely expressed^(^ ^19^ ^)^; its broad expression, along with that of CYP27B1, strongly implicates physiological roles of locally produced 1,25D acting in an intracrine or paracrine fashion. Among the cells that express CYP27B1 are activated macrophages and dendritic cells of the innate immune system.^(^ ^20^, ^21^, ^22^ ^)^ Importantly, contrary to the renal 1α‐hydroxylase, CYP27B1 activity in these cells is regulated by immune inputs such as IFN‐γ, a T‐cell cytokine secreted by proinflammatory Th1 cells, as well as agonists of PRRs such as the TLRs. The most critical finding that provides evidence that 1,25D signaling contributes to innate immunity emerged from a study in which Modlin and collaborators stimulated human macrophages with the 19‐kDa lipopeptide ligand of TLR1 and TLR2 (TLR2/1) heterodimers, which led to induction of *VDR* and *CYP27B1* expression and enhanced endogenous production of 1,25D from circulating 25D^(^ ^23^ ^)^ (Fig. 1). Likewise, triggering TLR4 receptors through lipopolysaccharide (LPS) signaling also upregulated *CYP27B1* expression^(^ ^22^ ^)^ (Fig. 1).

1,25D synthesis induced by TLR4 signaling is of interest given that we have known since the early 1990s that the gene encoding the TLR4 coreceptor, *CD14*, is a target gene of 1,25D.^(^ ^24^ ^)^ in vitro studies of CYP27B1 induction in cells of the monocyte lineage are compatible with the clinical observation of excessive 1,25D production by macrophages in patients with the granulomatous disease sarcoidosis.^(^ ^25^ ^)^ An intermediate in the pathway from TLR2/1 stimulation to the synthesis of 1,25D was later identified as being IL‐15; the cytokine likely induces CYP27B1 mRNA expression through the transcription factor C/EBP‐β.^(^ ^26^ ^)^ Because IL‐15 is an inducer of macrophage differentiation from monocytes, this finding also connects CYP27B1 expression to macrophage development. In addition, TLR2/1 signaling induces VDR expression, and the degree of induction was contingent on serum 25D levels.^(^ ^23^ ^)^ Responses were reduced or even absent in macrophages cultured from subjects deficient in vitamin D. In particular, 25D levels in African Americans were roughly 50% less than those of white Americans, consistent with previous studies reporting vitamin D deficiency in the former population based on reduced cutaneous vitamin D synthesis in darker skin.^(^ ^27^, ^28^ ^)^ Globally, these observations reveal the importance of vitamin D metabolism in macrophages, as well as the importance of vitamin D sufficiency in the initiation of local 1,25D signaling.

Further analyses have shown that there are multiple transcription factors that regulate *CYP27B1* expression. In silico promoter analyses identified numerous nuclear factor κB (NF‐κB) sites in the proximal *CYP27B1* promoter, which were confirmed by electrophoretic mobility shift assays.^(^ ^29^ ^)^ Cotransfection of NF‐κB p50 and p65 subunits was found to substantially decrease activity of a *CYP27B1* proximal promoter fragment, and treatment of human embryonic kidney 293 cells with an NF‐κB inhibitor yielded enhanced *CYP27B1* mRNA expression.^(^ ^29^ ^)^ In addition to directly regulating CYP27B1 activity, NF‐κB can indirectly modulate the enzyme's transcription through mutual repression with nuclear receptors such as the arylhydrocarbon receptor (AhR), the constitutive androstane receptor (CAR), and the glucocorticoid receptor (GR), as well as control its activity posttranscriptionally via induction of heme oxygenase or regulation of cytochrome P450 (CYP) protein stability.^(^ ^30^ ^)^ Another transcription factor that controls expression of the gene is C/EBP‐β (CCAAT/enhancer‐binding protein β); TLR2/1 stimulation by mycobacterial lipoprotein was found to trigger *CYP27B1* transcription by activating C/EBP‐β, which was critical for lipoprotein‐induced expression of the antimicrobial peptide cathelicidin and stimulation of autophagy.^(^ ^31^ ^)^ Functional putative CCAAT boxes were also found in the promoter of the CYP27B1 gene.^(^ ^32^ ^)^ Finally, two new putative transcription factors associated with *CYP27B1* transcriptional regulation in LPS‐challenged human mononuclear phagocytes were recently identified in silico: PLAGL2, a zinc finger protein that recognizes nucleic acids, and STAT4.^(^ ^33^ ^)^

## 1,25D Induces Antimicrobial Innate Immunity

The findings described above demonstrated that immune cells such as macrophages produce 1,25D upon pathogen intrusion. 1,25D through binding to the VDR, in turn, can regulate innate immune responses upstream and downstream of PRR signaling by activating transcription of the genes implicated in innate immunity. As mentioned previously, CD14, the coreceptor of TLR4 is robustly induced by 1,25D in human and murine cells.^(^ ^24^, ^34^ ^)^ Moreover, in human keratinocytes, vitamin D enhanced TLR2 expression, and considering that signaling through either TLR2 or TLR4 improves vitamin D signaling by boosting expression of the VDR and CYP27B1, the outcome of 1,25D signaling on TLR2 and CD14 expression in keratinocytes represents a positive feedback loop.^(^ ^34^ ^)^ Interestingly, this loop is not present in monocytes.^(^ ^35^ ^)^ 1,25D treatment of human monocytes inhibited both TLR2 and TLR4 mRNA and protein expression, and this regulation was found to be time‐ and dose‐dependent. However, CD14 expression was induced by 1,25D; it was hypothesized that downregulation of PRRs by vitamin D in antigen‐presenting cells may serve to suppress extreme Th1‐mediated inflammatory responses and downstream autoimmunity.^(^ ^35^ ^)^ In addition, human macrophages, in the presence of 1,25D, were found to restrict dengue virus infection by suppressing expression of the mannose receptor, which belongs to the C‐type lectin family of PRRs.^(^ ^36^ ^)^

Strong evidence implicating direct regulation of antimicrobial innate immune responses by 1,25D arose from the identification of vitamin D response elements (VDREs) adjacent to the transcription start sites of two genes that encode antimicrobial peptides (AMPs) β‐defensin 2 (DEFB2/DEFB4/HBD2) and cathelicidin antimicrobial peptide (CAMP/LL37)^(^ ^37^ ^)^ (Fig. 1). The study revealed a robust induction of *CAMP* by 1,25D in all tested cell types, which included multiple cell lines as well as human primary cell cultures; this initial finding was confirmed in subsequent in vitro and in vivo studies.^(^ ^23^, ^38^, ^39^ ^)^ Consistent with this, treatment with 1,25D stimulated secretion of antibacterial activity against pathogens such as the lung pathogen, *Pseudomonas aeruginosa* into conditioned media.^(^ ^37^ ^)^ As for *HBD2*, its expression by 1,25D alone was either modest or not detected.^(^ ^23^ ^)^ However, 1,25D was observed to enhance induction of *HBD2* by IL‐1β, a robust inducer of the antimicrobial peptide.^(^ ^37^ ^)^ Signaling through TLR2/1 pattern recognition receptors in human monocytes was later shown to upregulate IL‐1β expression and signaling; moreover, the combination of IL‐1β and 1,25D is required to drive potent induction of *HBD2*.^(^ ^40^ ^)^ It is likely that IL‐1β signaling stimulates binding of the NF‐κB transcription factor to tandem sites in the proximal *HBD2* promoter.^(^ ^40^ ^)^ Hence, the above findings suggest a molecular basis for vitamin D sufficiency providing broad protection against bacteria and viruses. For instance, *Helicobacter pylori* and rhinovirus infections enhance the expression of defensins^(^ ^41^, ^42^ ^)^ and human cathelicidin was demonstrated to inhibit HIV‐1 replication,^(^ ^43^ ^)^ indicating that 1,25D‐induced DEFB2 and CAMP expression may enhance protection against these pathogens (Fig. 1).

Interestingly, many underlying mechanisms of vitamin D signaling on innate immunity appear species‐specific. Although *CD14* expression induced by 25D was abolished in mice that lack CYP27B1,^(34^ ^)^ and macrophage *Cyp27b1* expression can be induced by TLR signaling or IFN‐γ in rodents,^(^ ^21^ ^)^ there is substantial evidence suggesting that many mechanisms of vitamin D‐regulated innate immunity are mechanistically conserved in primates only. For instance, the VDRE in the *CAMP* gene was shown to be present within an Alu repeat found exclusively in primates.^(^ ^38^ ^)^ The transposition event would appear to have occurred in a primate progenitor, and as a result, is evolutionarily conserved in humans, apes, and Old and New World monkeys.^(^ ^44^ ^)^ This species specificity is further highlighted by the conservation of VDREs in *HBD2*, *CAMP*, and *NOD2* genes in primates, but not in rodents.^(^ ^45^ ^)^ Furthermore, although the *HBD2*, *CAMP*, and *NOD2* genes are induced by 1,25D in human cells of epithelial or myeloid origin, they are not similarly regulated in murine cells. Likewise, conditioned media from 1,25D‐treated mouse epithelial cells did not generate antimicrobial activity against *Escherichia coli* nor *P. aeruginosa*, whereas the activity was produced against both bacterial types in human epithelial cells exposed to 1,25D.^(^ ^45^ ^)^

Distinct mechanisms of regulation of AMP expression in humans and mice have been proposed to be caused by mice being nocturnal, whereas humans are active during daytime and can consequently acquire vitamin D from exposure to sunlight.^(^ ^23^ ^)^ It is also possible that some differences in human versus murine immune function are caused by different sites for synthesis of 1,25D, with some reports suggesting that CD8+ T cells rather than macrophages are the major immune cell source of CYP27B1 expression and activity in mice.^(^ ^46^ ^)^ Despite these key differences between humans and mice, it is important to recognize that there is also significant homology in immune responses to vitamin D. This is particularly evident in comparisons of human and mouse models of immune disease. Recent studies have shown similarities between human and murine models of infectious disorders such as sepsis,^(^ ^47^ ^)^ suggesting that there is some commonality in innate immune response to infection between species, including dysregulation of the murine CAMP homolog, cathelicidin‐related antimicrobial protein (CRAMP) in vitamin D‐deficient mice.^(^ ^47^ ^)^ This study also highlighted another key facet of species homology in immune responses to vitamin D, namely the link between vitamin D deficiency and dysregulated inflammation in humans and mice. Indeed, several mouse models of inflammation are known to replicate human diseases, notably autoimmune diseases such as MS and inflammatory bowel disease (IBD),^(^ ^48^ ^)^ where anti‐inflammatory T‐cell responses to 1,25D show similarity to human T‐cell responses.

1,25D also regulates the expression of another antibacterial agent, hepcidin antibacterial protein (HAMP; Fig. 1).^(49)^ In contrast to CAMP and DEFB4, the direct microbiocidal properties of HAMP are not as apparent. Rather, the major function of hepcidin is to suppress ferroportin‐mediated export of iron, and it plays a role in the anemia of infection or inflammation. Almost all microorganisms use iron to sustain their growth; therefore, restricting circulating levels of iron presents a significant host response to systemic infection.^(^ ^50^ ^)^ Moreover, bacterial and viral stimuli are known to enhance the expression of HAMP.^(^ ^51^ ^)^ Therefore, in this context, suppression, rather than induction, of HAMP by vitamin D may be of benefit because by abrogating HAMP‐induced suppression of ferroportin, this facilitates iron export and lower intracellular concentrations of iron. Indeed, Hewison and colleagues have shown that once bound to the VDR, the active hormone transcriptionally suppresses the *HAMP* gene in monocytes and hepatocytes.^(^ ^49^ ^)^ This led to a corresponding increase in the membrane expression of ferroportin and a decrease in ferritin expression, which serves as a biomarker for intracellular iron levels. Furthermore, in contrast to CAMP and DEFB4, elevated serum 25D levels subsequent to vitamin D supplementation of human subjects were correlated with a decrease in the circulating concentrations of HAMP. However, similar to cathelicidin and defensin, 1,25D directly regulates hepcidin gene expression by binding to specific VDREs embedded in the *HAMP* promoter, and its regulation is not observed in murine models, suggesting that control of the HAMP‐ferroportin axis by vitamin D is part of the same evolutionary adaptations as those of the AMPs described earlier in this review (Fig. 1).^(49)^

## 1,25D Regulates Pattern Recognition Receptor Gene Expression

Another aspect of innate immune signaling regulated by vitamin D is PRR expression. In addition to CD14, expression of the intracellular pathogen‐sensing protein NOD2/CARD15/IBD1 is transcriptionally induced by 1,25D in cells of monocytic and epithelial origin.^(^ ^52^ ^)^ Two distal VDREs were located in the NOD2 gene, and the function of the putative VDREs were confirmed by chromatin conformation capture assay, which identifies loops between distant chromatin sites that can be viewed by the formation of ligation‐dependent PCR products. NOD2/CARD15 recognizes muramyl dipeptide (MDP), a lysosomal breakdown product of bacterial peptidoglycan and in cells expressing functional NOD2, cotreatment with 1,25D and MDP displayed synergistic NF‐κB‐dependent induction of DEFB4 and CAMP (Fig. 1). However, the regulation was absent in cells from patients with Crohn disease (CD) that were homozygous for a nonfunctional mutation of the *NOD2* gene.^(^ ^52^ ^)^ Notably, these findings provide a strong association between 1,25D signaling and the pathogenesis of CD, a relapsing IBD originating from insufficient management of intestinal bacterial load by innate immune cells.^(^ ^53^ ^)^ Susceptibility to CD has been linked with polymorphisms in both the *NOD2* and *HBD2* loci,^(^ ^54^, ^55^ ^)^ and although vitamin D deficiency has been associated with CD, there is some disagreement as to whether the deficiency arises from intestinal malabsorption of the hormone or whether the insufficiency contributes to pathogenesis of the disease. However, the direct induction of *NOD2* expression, as well as the direct and indirect regulation of *HBD2* expression, suggests a molecular link between 1,25D deficiency and CD. Furthermore, 1,25D‐mediated regulation of NOD2 may be relevant to fighting intracellular infections by mycobacteria (eg, *Mycobacterium tuberculosis*), as these bacteria generate the N‐glycolyl form of MDP, which binds to NOD2 with higher affinity than the N‐acetyl form^(^ ^56^ ^)^ (Fig. 1).

The identification of NOD2 as a 1,25D target gene also provides a connection between 1,25D and autophagy, a crucial defense mechanism involving the usage of autophagosomes to target intracellular pathogens for lysosomal degradation.^(^ ^57^ ^)^ In particular, MDP‐bound NOD2 recruits the autophagy protein ATG16L1, the product of a CD susceptibility locus, to the plasma membrane at the entry site of bacteria and, upon stimulation, induces autophagy and clearance of pathogen. However, in cells homozygous for the CD‐associated NOD2 and ATG16L1 mutations, this effect was abolished, indicating that impaired autophagy in cells with these mutations supports their contribution to the pathogenesis of CD.^(^ ^57^ ^)^ In addition, there is evidence for 1,25D‐induced expression of CAMP and enhanced autophagy in macrophages infected with mycobacteria.^(^ ^58^ ^)^ Direct effects on autophagy by vitamin D have also been reported; for instance, synthetic 1,25D analogs were shown to inhibit the mammalian target of rapamycin (mTOR), which is known to result in autophagy.^(^ ^59^ ^)^ Also, more recently, the VDR was documented as a master transcriptional regulator of autophagy in breast cancer cells.^(^ ^60^ ^)^

## 1,25D Regulates Expression of Cytokines Important in Innate Immunity

The induction of genes encoding cytokines and chemokines also represents a crucial part of 1,25D‐mediated innate protection against pathogen threat; this was highlighted by transcriptome profiling of *M. tuberculosis (Mtb)* infected and noninfected macrophages in the presence or absence of 1,25D, which revealed numerous genes regulated by 1,25D being altered by infection.^(^ ^61^ ^)^ Among those genes were several chemokines and cytokines, and 1,25D was found to broadly enhance the infection‐stimulated chemokine/cytokine response. Specifically, 1,25D directly augmented transcription of IL‐1β, one of the first cytokines generated in response to infection, and is released into the circulation from its pro‐form by proteolytic cleavage catalyzed by caspase 1 coupled to PRR‐activated complex known as the inflammasome (Fig. 1). The physiological significance of this was demonstrated by a coculture of infected macrophages with primary human airway epithelial cells; from this, it was shown that the 1,25D and *Mtb* induced IL‐1β secretion, prolonged survival in infected macrophages, and decreased *Mtb* burden in those cells by inducing DEFB4 production in the cocultured lung epithelial cells. IL‐8, a neutrophil chemoattractant, is another cytokine robustly stimulated by 1,25D in uninfected and *Mtb‐*infected macrophages, and it would be of interest to investigate whether there is a corresponding effect on the expression of CXCR2, the IL‐8 cell surface receptor, on 1,25D‐treated pathogen‐challenged human neutrophils (Fig. 1). Other secreted agents generated by 1,25D‐exposed infected macrophages include the chemokines CCL3, CCL4, and CCL8.^(^ ^61^ ^)^

1,25D has also been shown to dose‐dependently attenuate the expression of proinflammatory cytokines IL‐6, TNFα, and IFN‐γ in *Mtb‐*infected human peripheral blood mononuclear cells.^(^ ^62^ ^)^ On the other hand, the authors of this study also reported enhanced expression of IL‐10, an anti‐inflammatory cytokine, by 1,25D. The proposed mechanism behind the inhibited proinflammatory cytokine response appears to involve a VDR‐mediated repression at the mRNA and protein level of the PRRs TLR2, TLR4, Dectin‐1, and mannose receptor.^(^ ^62^ ^)^ Furthermore, LPS‐induced production of IL‐6 and TNFα was inhibited by 1,25D in human and murine monocytes and macrophages,^(^ ^63^, ^64^ ^)^ and this regulation appeared to differ with the stage of monocyte/macrophage maturation and was dependent on MAPK phosphatase 1.^(^ ^64^ ^)^ There is also evidence for 1,25D acting in synergy with other agents to regulate IL‐6 expression. For example, 1,25D was found to enhance glucocorticoid‐induced suppression of LPS‐stimulated IL‐6 in human monocytes.^(^ ^65^ ^)^

## Vitamin D and Granulocyte Biology

Most of the studies to date have investigated the innate immune regulation by vitamin D in monocytes and macrophages. However, it is important to note that a variety of cell types express PRRs and acquire the necessary tools to generate innate immune responses to pathogen threat. The most abundant among these are granulocytic cells such as neutrophils; these short‐lived cells with a circulating half‐life of 6–8 hours represent 70% of all leukocytes, and in response to inflammatory stimuli, are rapidly recruited to sites of infection where they efficiently bind, engulf, and inactivate bacteria.^(^ ^66^ ^)^ Neutrophils are known to express VDR mRNA at a level comparable to monocytes, and once treated with 1,25D, also induce expression of CD14.^(^ ^67^ ^)^ These granulocytic cells are among the cell types that exhibit 1,25D‐driven expression of CAMP, although the physiological relevance of this has yet to be determined.^(^ ^37^ ^)^ Unlike monocytes, however, neutrophils do not appear to express CYP27B1, suggesting that they are more likely to be systemic responders to hormonal 1,25D. Despite this, they appear to be the major source of serum cathelicidin/LL37 based on their abundance and the presence of neutrophil granules that store the majority of LL37 released at infection sites.^(^ ^68^, ^69^ ^)^ A link between low serum LL37 and mortality in patients with chronic kidney disease has been reported; the levels of LL37 correlated with serum 1,25D rather than 25D, which may serve as an endocrine‐regulated innate immune response that may involve CYP27B1‐negative, VDR‐positive neutrophils.^(^ ^70^, ^71^ ^)^ However, in patients with sepsis, a systemic inflammatory disease associated with maintained presence of neutrophils, circulating levels of LL37 are lower in more severe cases, and this is correlated with low serum levels of 25D.^(^ ^72^ ^)^

In the early 2000s, a physiological role for the VDR in neutrophils was investigated by using differential display analysis to identify expression of genes in 1,25D‐treated, LPS‐stimulated neutrophils.^(^ ^67^ ^)^ Of the genes identified, the neutrophil elastase inhibitor trappin‐2/elafin/SKALP was potently induced in LPS‐exposed neutrophils, but was mildly suppressed by 1,25D. Under the same conditions, IL‐1β was slightly inhibited by the active hormone.^(^ ^67^ ^)^ Since then, a few other studies have delved into the role of vitamin D in neutrophil innate immunity. Among those was one that found increased apoptosis in neutrophils from patients with chronic obstructive pulmonary disease (COPD) treated with 1,25D.^(^ ^73^ ^)^ This is noteworthy because COPD pathogenesis is characterized by a lower rate of neutrophil apoptosis, and vitamin D acquired the capacity to counter this via activation of p38 MAPK. Furthermore, 1,25D treatment enhanced the production of IL‐8 in LPS‐exposed neutrophils; however, no effect was detected on the phagocytic capacity of the cells when challenged with *E. coli*.^(^ ^74^ ^)^

Recently, the formation of neutrophil extracellular traps (NETs) by vitamin D was explored; the production of these networks of extracellular fibers composed of DNA, histones, and enzymes that function to immobilize pathogens were enhanced by 1,25D.^(^ ^75^ ^)^ However, the NETs‐like structures were not verified to be bona fide NETs. Moreover, 1,25D was reported to augment neutrophil killing of *Streptococcus pneumoniae* and also to lower inflammatory cytokine production by inducing the expression of the anti‐inflammatory cytokine IL‐4 and suppressor of cytokine signaling (SOCS) proteins.^(^ ^76^ ^)^ Overall, these findings suggest a role for vitamin D in dampening neutrophil‐driven inflammatory responses, while still boosting pathogen killing by the cells; however, one study found that although 1,25D decreased LPS‐induced expression of macrophage inflammatory protein‐1β and VEGF in adult neutrophils, this anti‐inflammatory response was not observed in neonatal cells.^(^ ^77^ ^)^ The authors speculate that this may be because of the decreased expression of VDR and CYP27B1 in neonates.

Eosinophils, granulocytic effector cells involved in T‐helper type 2 cell‐ (Th2‐) mediated diseases, such as asthma and atopic dermatitis,^(^ ^78^ ^)^ are also subject to the effects of vitamin D. In a murine model of lung eosinophilic inflammation, treatment with vitamin D decreased airway eosinophilia, and this was attributed to the downregulatory effect of 1,25D on IL‐15, the key cytokine implicated in recruitment of eosinophils to local inflammatory sites.^(^ ^79^ ^)^ Likewise, vitamin D supplementation was found to reduce eosinophilic airway inflammation in patients with nonatopic asthma and severe eosinophilic airway inflammation in a randomized controlled trial.^(^ ^80^ ^)^ Another cell type well‐known for its role in allergic responses is the mast cell, which expresses the *VDR* and, unlike neutrophils, also expresses *CYP27B1*.^(^ ^81^ ^)^ Both 25D and 1,25D were demonstrated to suppress IgE‐mediated mast‐cell derived proinflammatory and vasodilatory mediator production in human and murine mast cells. In addition, in response to 1,25D, production of the immune regulatory IL‐10 is significantly upregulated in mouse mast cells,^(^ ^82^ ^)^ providing an explanation for why mice deficient in mast cells are resistant to the immune suppressive effects of UVB.^(^ ^83^ ^)^ UVB irradiation transmits the immune suppressive signal in the skin by triggering mast cells to migrate to lymph nodes.^(^ ^84^ ^)^ There, the IL‐10‐producing mast cells contact B cells and inhibit antibody class‐switch recombination and affinity maturation in germinal centers. Because mast cells are known to induce activation of T regs and B cells, it is speculated that this is a mechanism by which UVB and vitamin D are working to repress some adaptive immune responses.^(^ ^84^ ^)^

## Effects of Vitamin D on NK, NKT, and γδ T Cells

Natural killer (NK) cells acquire the capacity to efficiently kill infected cells and, because some NK subsets can modulate adaptive immune responses by the secretion of cytokines and chemokines, they are generally described to function at the interface between innate and adaptive immunity.^(^ ^85^ ^)^ Despite some studies showing no effect of 1,25D on NK function,^(^ ^86^, ^87^, ^88^ ^)^ others suggest an important role for 1,25D on NK cell biology.^(^ ^89^, ^90^, ^91^, ^92^, ^93^, ^94^ ^)^ Patients with diseases that affect metabolism of vitamin D, such as chronic renal failure and vitamin D‐resistant rickets, have impaired activity of NK cells.^(^ ^89^, ^90^ ^)^ In these patients, vitamin D supplementation can improve and even normalize NK‐cell activity. In a healthy elderly population, it was shown that NK‐cell cytolytic function is associated with serum 25D levels.^(^ ^93^ ^)^ This was later confirmed in in vitro studies, where 1,25D treatment dose‐dependently enhanced the activity of NK cells without influencing cell proliferation by increasing cellular granule content and shortening the delay in the secretion of granzyme A.^(^ ^91^, ^94^ ^)^ Furthermore, 1,25D in combination with the synthetic glucocorticoid dexamethasone induced IL‐10 mRNA expression in NK cells, implying that vitamin D signaling can stimulate a regulatory phenotype, where antigen‐specific T‐cell responses are suppressed, in NK cells.^(^ ^92^ ^)^ Recently, one study noted that 1,25D had regulated cytotoxicity, cytokine secretion, and degranulation in NK cells isolated from women with idiopathic recurrent pregnancy losses, a condition associated with aberrant NK activity and vitamin D deficiency.^(^ ^95^ ^)^ Other current reports also corroborate a regulatory role for vitamin D in NK‐cell effector function.^(^ ^96^, ^97^ ^)^

CD1d‐reactive natural killer T (NKT) cells, a subset of T cells that lack immunological memory,^(^ ^98^ ^)^ are thought to be among the most immediate producers of cytokines in the innate immune response.^(^ ^99^ ^)^ As a result of early IL‐4 production by activated NKT cells, NKT cell activation delays the onset and represses the symptoms of experimental autoimmune encephalomyelitis and experimentally induced colitis.^(^ ^100^, ^101^ ^)^ Furthermore, low numbers of NKT cells are correlated with increased susceptibility to autoimmunity.^(^ ^102^ ^)^ Although the role of vitamin D in NKT cell biology is not yet completely elucidated, animal studies on the topic have revealed differing roles for the VDR and 1,25D in the regulation of invariant NKT cell (iNKT) numbers and function. *VDR* gene knock‐out (KO) mice demonstrate a blockade in iNKT cell development, resulting in reduced numbers of NKT cells in the thymus, liver, and spleen compared with their wild‐type (WT) counterparts.^(^ ^103^ ^)^ Furthermore, the residual iNKT cells from VDR KO mice are intrinsically defective, and only a small fraction of the cells produce IL‐4 and IFN‐γ. In contrast, 1,25D sufficiency appears to only regulate the number of iNKT cells, as evidenced by decreased iNKT cell counts in vitamin D‐deficient mice in utero.^(^ ^104^ ^)^ Remarkably, this was found to not be corrected by subsequent intervention with 1,25D based on increased apoptosis of early thymic iNKT cell precursors. From these findings, it appears that the quantity of vitamin D available in the environment during prenatal development may influence the number of iNKT cells and the subsequent resulting risk of autoimmunity.^(^ ^104^ ^)^

Arguably the most intricate and advanced cellular representative of the innate immune system are γδ T cells.^(^ ^105^ ^)^ Representing a small number of T cells in the circulation of healthy individuals, an increase in the number and inflammatory phenotype of γδ T cells has been detected in patients with autoimmune hepatitis.^(^ ^106^, ^107^ ^)^ Interestingly, transcriptional profiling of γδ T cells revealed an upregulation of VDR upon activation of these cells with phosphate‐containing ligands.^(^ ^108^ ^)^ Activity of the receptor was demonstrated by observing a 1,25D‐mediated dose‐dependent inhibition of phospho‐ligand‐induced γδ T‐cell expansion and IFN‐γ production. Similarly, in cattle, 1,25D production by macrophages within TB lesions inhibited proliferation of γδ T cells and decreased expression of the activation marker CD44 on the surfaces of remaining responding cells.^(^ ^109^ ^)^ These studies globally propose an immunosuppressive role of vitamin D on this small subset of T cells as part of the more generalized anti‐inflammatory response to 1,25D.

## Vitamin D and T Cells

Although primarily an activator of the innate immune system to enhance immediate response to infection, vitamin D acts to regulate activity of the adaptive immune system. Consistently, low levels of circulating serum 25D have been correlated with increased risk of developing T‐cell‐mediated autoimmune diseases such as MS,^(^ ^110^, ^111^ ^)^ T1DM,^(^ ^112^ ^)^ IBD,^(^ ^113^ ^)^ systemic lupus erythematous (SLE),^(^ ^114^ ^)^ and rheumatoid arthritis (RA).^(^ ^115^ ^)^ These associations may also correlate with exposure to UV light;^(^ ^112^, ^114^ for example, patients with SLE experience increased sensitivity to UV light, which leads to avoidance.^(^ ^114^ ^)^ However, despite these observations and the well‐described direct and indirect effects of 1,25D on T cells as detailed below, therapeutic use of vitamin D in supplementation trials has so far shown only modest effects. There remains a need to better understand the mechanisms of 1,25D‐mediated immunomodulation to improve treatment of autoimmune inflammatory disease.

### VDR expression and T‐cell activation

Early studies showed that the intracellular VDR, as determined by specific 1,25D binding, was expressed in activated lymphocytes.^(^ ^116^ ^)^ Since then, levels of VDR expression on T‐cell subsets have been investigated further, occasionally with conflicting results. As 1,25D acts primarily through binding to the VDR, it is important to consider VDR expression levels as a determinant of T‐cell function. Studies using VDR knockout (KO) mice have shown comparable levels of CD4^+^ and CD8αβ^+^ T cells in the periphery, indicating that 1,25D is not required for T‐cell thymic development or subsequent egress from the thymus.^(^ ^117^ ^)^ This is with the exception of iNKT cells and CD8αα^+^ T cells, a protective subset found in the gut, which fail to develop from their thymic precursors in VDR KO mice.^(^ ^103^, ^118^ ^)^ With no overall abnormalities in peripheral T‐cell numbers, it appears that 1,25D acts more as a regulator of fully developed and differentiated cells, to dampen overactive responses, than a regulator of development.

**Fig 2:**
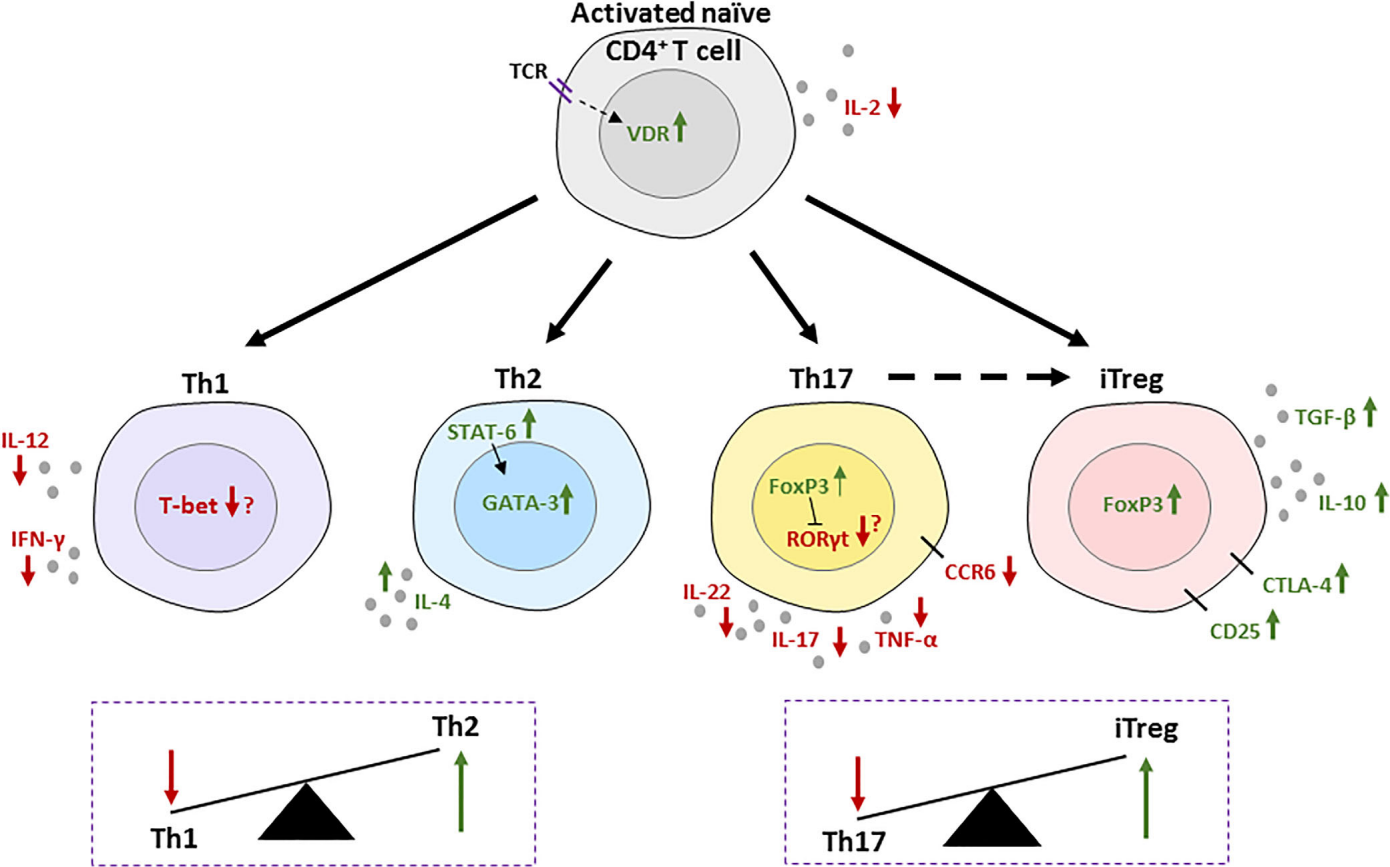
Immunomodulatory effects of 1,25D on CD4^+^ T cells. T‐cell‐receptor signaling induces an upregulation in the vitamin D receptor (VDR), which is stabilized from degradation by 1,25D binding. IL‐2 production is suppressed by 1,25D signaling after T‐cell activation. Th1‐ and Th17‐cell differentiation is suppressed by vitamin D_3_ signaling along with characteristic, inflammatory cytokine production. CCR6 on Th17 cells is also suppressed, preventing homing to tissues. Downregulation of transcription factors T‐bet and RORγt is less consistently reported. Th2‐cell differentiation is widely thought to be increased by 1,25D, although this is dependent on conditions. 1,25D causes upregulation of IL‐4 and STAT‐6, which increases GATA‐3. Overall, the Th1/Th2 balance is in the favor of Th2 cells. Similarly, the Th17/iTreg balance favors Tregs with the addition that Th17 cells, additionally suppressed by FoxP3 upregulation, can convert to a Treg phenotype. 1,25D upregulates IL‐10, TGF‐β, and inhibitory markers CTLA‐4 and CD25 to promote an anti‐inflammatory phenotype.

VDR is expressed at very low,^(^ ^119^ ^)^ and often undetectable,^(^ ^120^, ^121^ ^)^ levels in naïve T cells. Upon activation, in response to T‐cell receptor (TCR) signaling via the p38 MAPK pathway, expression of the VDR is significantly increased in T cells.^(^ ^119^, ^120^ ^)^ One study suggested that this initial upregulation of the VDR then led to the upregulation of PLCγ‐1, a key enzyme in T‐cell activation, which was able to reciprocally further enhance TCR signaling.^(^ ^119^ ^)^ It is also reported that 1,25D upregulates expression of the T‐cell markers CD38 and HLA‐DR.^(^ ^122^ ^)^ However, these studies were carried out in vitro and require further investigation in vivo to fully define the relevance of this finding. In comparison, mice have sufficient levels of PLCγ‐1 prior to TCR activation and do not require upregulation of the enzyme.^(^ ^123^ ^)^ Binding of 1,25D to the VDR has been shown to prevent degradation of the receptor, forming a positive feedback loop for increased VDR levels in activated T cells (Fig. 2).^(^ ^120^ ^)^

### VDR expression in T‐cell subsets

Although it is known that all naïve T cells upregulate VDR expression upon TCR activation, there are conflicting reports on the level of VDR expression in differentiated T‐cell subsets. In one study, human naïve CD4^+^ T cells differentiated for 3 days in the presence of cytokines that induced Th1, Th17 or Th2 cells were shown to have equal levels of VDR in all three Th types (120). However, it is likely that 3 days is not sufficient time for full polarisation of human CD4^+^ T cell subsets and therefore VDR expression in this case may reflect the initial upregulation of the receptor after TCR engagement. In mice, studies have reported that Th1 cells do not express the VDR to the same level as Th2 or Th17 cells,^(^ ^124^, ^125^ ^)^ even suggesting levels to be comparable to naïve T cells.^(^ ^124^ ^)^ The latter of these studies saw no change in IFN‐γ with 1,25D treatment,^(^ ^125^ ^)^ an effect that is usually observed in vitro.^(^ ^126^, ^127^ ^)^ There have been similar reports for induced regulatory T cells (iTregs). One study, sorting human Tregs as CD4^+^CD25^hi^ cells, showed comparable expression of the VDR in Tregs and T conventional cells.^(^ ^128^ ^)^ However, because CD25 is upregulated during T‐cell activation, it is possible there was some contamination of the Treg population during sorting. In mice, it was shown that Tregs have much lower levels of VDR expression compared with Th2 and Th17 cells.^(^ ^124^, ^129^ ^)^ The potential functional impact of differences in VDR expression levels between T‐cell subsets, or between human and mouse, remains unclear.

### VDR polymorphisms and inflammatory disease

In addition to the relationship between circulating 25D status and risk of T‐cell‐mediated inflammatory diseases described above, research has also implicated changes in VDR structure and function with susceptibility to autoimmune disease. Genome‐wide association studies (GWASs) have identified multiple single nucleotide polymorphisms (SNPs) that increase susceptibility to a disease. In such studies, four main polymorphisms in the *Vdr* gene: *Taq*I, *Bsm*I, *Apa*I, and *Fok*I have been linked to increased susceptibility to MS, TIDM, IBD, SLE, and RA.^(^ ^130^, ^131^, ^132^, ^133^, ^134^, ^135^ ^)^ Association of the four *VDR* variants differs between population and disease, but the presence of SNPs in the *VDR* gene is common to GWASs of many T‐cell‐mediated inflammatory diseases.

Alongside these GWASs, others directly link VDR abnormalities in T cells to increased risk of autoimmune inflammatory disease. For example, one study demonstrated that a promoter region of the *VDR* gene had increased levels of methylation in T cells from patients with MS compared with healthy controls.^(^ ^136^ ^)^ Additionally, in both cell lines and human T cells, when cells were cultured in the presence of low serum 25D, it was shown that there was decreased binding of the VDR to multiple gene variants commonly identified in GWASs to predispose to autoimmune disease.^(^ ^137^ ^)^ The above studies highlight the complex nature of disease susceptibility, but strongly suggest a role for vitamin D in limiting the pathogenicity of T‐cell‐mediated autoimmune diseases.

## Direct Effects of 1,25D on T Cells

### IL‐2 production and proliferation

The regulatory effects of 1,25D can be seen almost immediately after T‐cell activation with the inhibition of IL‐2 production (Fig. 2).^(^ ^138^ ^)^ The *IL2* gene has two VDR response elements,^(^ ^139^ ^)^ with another demonstrating that the VDR‐RXR heterodimer prevents the formation of the NFAT/AP‐1 complex, which is required for activation of the *IL2* promoter.^(^ ^140^, ^141^ ^)^ Initial studies showed that 1,25D inhibited T‐cell proliferation.^(^ ^138^, ^142^ ^)^ This was observed in bulk peripheral blood mononuclear cells (PBMCs) and purified populations of CD4^+^ or CD8^+^ T cells.^(^ ^138^, ^142^, ^143^ ^)^ Recently, transcriptome analysis of CD4^+^ T cells from mice with experimental autoimmune encephalomyelitis (EAE), a model of MS, treated with vitamin D showed downregulation of multiple genes involved in proliferation.^(^ ^144^ ^)^ The full antiproliferative effects of 1,25D are not completely understood; however, several potential mechanisms have been proposed.

In cancer cells, it has been reported that 1,25D causes cells to arrest at the G1 phase of the cell cycle by preventing cyclin‐dependant kinase activity, inhibiting proliferation.^(^ ^145^ ^)^ In EAE, 1,25D treatment decreased expression of cyclins in CD4^+^ T cells.^(^ ^144^ ^)^ However, other evidence for cell‐cycle alteration by 1,25D in T cells is limited. Some studies have suggested that the antiproliferative effects of 1,25D are attributable to the reduction in IL‐2 production^(^ ^138^, ^142^ ^)^ and others have linked it to the effects of 1,25D on antigen‐presenting cells, thereby indirectly suppressing T‐cell proliferation.^(^ ^126^, ^146^ ^)^ In one study, cultured purified CD4^+^CD25^−^ T cells in the presence of 1,25D demonstrated no changes in proliferative capacity, suggesting that the antiproliferative effects seen previously were caused by the actions of other cells in the environment.^(^ ^126^ ^)^ A few reports have suggested that 1,25D is able to sensitize T cells to undergo apoptosis in EAE^(^ ^147^ ^)^ and a T1DM mouse model,^(^ ^148^ ^)^ but the relevance of this effect for disease pathology has still to be determined.

### Th1 cells

In both human and mouse studies, 1,25D has been shown to inhibit Th1 and reciprocally promote Th2 cell differentiation.^(^ ^79^, ^127^, ^149^, ^150^ ^)^ In these studies, the change in the T‐cell subset is described as an alteration in cytokine profile. This is often a decrease in the Th1‐cell‐associated cytokine, IFN‐γ, and an increase in Th2‐cell cytokines such as IL‐4, but sometimes also IL‐5 and IL‐13. Transcription factors appear to be less consistently affected, although changes in T‐bet (*Tbx21*) and *Gata3* expression are reported (Fig. 2).^(^ ^151^ ^)^ Although described in multiple studies as a Th1‐to‐Th2 skew, inhibition of Th1 cell differentiation is not always accompanied by an increase in Th2 cells.^(^ ^151^, ^152^ ^)^ In addition to these effects on Th differentiation, 1,25D is also reported to directly inhibit IFN‐γ expression in existing Th1‐effector cells.^(^ ^126^, ^153^ ^)^

Mechanistically, it was demonstrated in one study that VDR‐RXR binds to promoter regions of the *Ifng* gene to directly inhibit transcription.^(^ ^154^ ^)^ However, it is likely that there are other mechanisms of IFN‐γ inhibition by 1,25D, as mRNA is not consistently downregulated in studies where downregulation of the protein is seen.^(^ ^150^ ^)^ IL‐12, an important cytokine for Th1 differentiation and expression of IFN‐γ, is also inhibited by 1,25D.^(^ ^152^ ^)^ It is likely that the decrease in IL‐12 partially limits the induction of a Th1 phenotype. This is demonstrated in two EAE studies, both highlighting the reduction in IL‐12 as key in preventing Th1‐cell development and ameliorating EAE.^(^ ^155^, ^156^ ^)^ Moreover, some studies do not demonstrate a decrease in IFN‐γ during Th1‐cell polarization in the presence of 1,25D, often these cultures contain supplemented IL‐12, which may partially reduce the inhibitory effects of 1,25D. This is shown in one study, where the addition of IL‐12 to 1,25D‐treated‐T cells increased IFN‐γ production to a level between control and 1,25D alone.^(^ ^157^ ^)^

### Th2 cells

It is widely considered that Th2 cells are increased by 1,25D, as a consequence of the transcriptional increase of GATA3, seen in multiple mouse and human studies.^(^ ^127^, ^149^, ^158^ ^)^ This is accompanied by an increased expression of the characteristic Th2 cytokine IL‐4; some studies also reported increased IL‐5 and IL‐13.^(^ ^127^, ^150^ ^)^ It has been suggested that the increase in GATA‐3 is downstream of upregulated STAT‐6 activity, mediated by 1,25D, although the mechanism for this is unclear.^(^ ^150^ ^)^ Th2 cells are unique in that they are both induced by and producers of the same cytokine, IL‐4. IL‐4 is able to induce STAT‐6, which then induces GATA‐3 and production of IL‐4 (Fig. 2).^(^ ^150^ ^)^

Literature on the effect of 1,25D on Th2 cells is, however, largely inconsistent, with differing effects between mouse, human, tissue, and culture methods. One study showed that there was only an increase in IL‐4 when T cells from human PBMCs were cultured in the presence of 1,25D and IL‐4, suggesting a requirement for the presence of IL‐4 in 1,25D‐mediated effects on Th2 differentiation.^(^ ^157^ ^)^ This is also highlighted by a polarization study on murine CD4^+^ T cells from lymphoid tissue, where IL‐4 was only increased in the cultures supplemented with IL‐4 or no polarizing conditions.^(^ ^153^ ^)^ However, in another study using Th2 polarizing conditions on murine naïve and memory CD4^+^ T cells from splenocytes, there was a decrease in IL‐4 production in naïve CD4^+^ T cells and no change in the memory CD4^+^ T cells.^(^ ^151^ ^)^ A study, which cultured human cord blood cells in the presence of 1,25D and IL‐4, found a decrease in IL‐4 production when compared with the cells cultured with IL‐4 alone.^(^ ^159^ ^)^ There is also often no change in IL‐4 in 1,25D‐treated mouse models of inflammation.^(^ ^160^, ^161^ ^)^ These variable observations are well‐demonstrated in one study in which IL‐4 was increased by 1,25D in murine T‐cell cultures, whereas in vivo, no change in IL‐4 was seen.^(^ ^79^ ^)^

### Th17 cells

Th17 cells are a unique subset of T cells characterized by their ability to produce IL‐17 and IL‐22, and express the transcription factor RORγt.^(^ ^58^ ^)^ Vitamin D acts on Th17 cells to suppress expression of IL‐17, IL‐22, TNF‐α, IFN‐γ, and the chemokine receptor CCR6, thereby preventing migration of Th17 cells to inflamed tissues (Fig. 2).^(^ ^124^, ^126^, ^158^, ^162^ ^)^ This inhibition of a Th17 phenotype has been reported following differentiation of naïve CD4^+^ T cells and ex vivo human Th17 memory cells.^(^ ^158^, ^162^ ^)^ Similar to Th1 cell suppression, data for effects of 1,25D on RORγt expression are inconsistent. One study using isolated human CD4^+^ memory T cells showed a reduction in cytokine transcription, but no change in RORγt with 1,25D treatment.^(^ ^162^ ^)^ In contrast, other studies in vivo and during in vitro Th17‐cell differentiation observed a reduction in RORγt mRNA, suggesting that 1,25D has a greater influence on transcription factors before full polarization, when T cells have a less‐stable phenotype.^(^ ^144^, ^153^, ^163^ ^)^

It has been well‐described that a reduction in Th17 cells often occurs in parallel to an increase in Tregs, as part of a proposed Th17/Treg reciprocation axis. An important focus of improving treatments for autoimmune diseases is to restore the Th17/Treg imbalance that occurs during chronic inflammation. Many studies analyzing the effect of 1,25D on Th17 cells have also reported an increase in Treg markers such as CTLA‐4, CD25, FoxP3, and in production of Treg cytokines such as IL‐10 (Fig. 2).^(^ ^126^, ^162^, ^164^ ^)^ The mechanism of action of 1,25D on Th17 cells is not completely understood, but some pivotal pathways have been identified. Expression of the Treg marker FoxP3 is directly upregulated by 1,25D, through binding of VDR to a VDRE in the *FOXP3* gene.^(^ ^165^, ^166^ ^)^ FoxP3 is able to suppress the upregulation of RORγt in CD4^+^ T cells,^(^ ^167^ ^)^ suggesting that by inducing FoxP3 expression in naïve CD4^+^ or Th17 cells, 1,25D is able to suppress Th17 differentiation.^(^ ^166^ ^)^ Additionally, 1,25D‐mediated inhibition of the NFAT pathway blocks the production of IL‐17,^(^ ^166^ ^)^ and upregulation of PLCγ‐1 by 1,25D induces the production of TGF‐β, further promoting the differentiation of Tregs.^(^ ^168^, ^169^ ^)^

### T‐regulatory cells

When reviewing the effects of 1,25D on Tregs, it is important to distinguish effects on Treg differentiation and plasticity from direct effects on Treg function. Evidence suggests that 1,25D is able to induce FoxP3 expression in naïve CD4^+^ T cells and promote Treg differentiation, with resulting functional increases in expression of IL‐10 and other regulatory markers such as CTLA‐4 (Fig. 2).^(^ ^126^, ^165^, ^169^ ^)^ Studies have also shown plasticity of Th17 and Th2 cells converted to a regulatory phenotype by 1,25D.^(^ ^162^, ^170^ ^)^ As highlighted above, induction of a Treg phenotype by 1,25D is attributed to the direct upregulation of genes such as *FOXP3* and *CTLA4* and parallel downregulation of genes such as *IFNG* and *IL17A*.^(^ ^126^, ^165^, ^166^, ^168^ ^)^ There is little evidence for direct induction of IL‐10 by 1,25D in T cells. However, in monocytes, VDR binding to a promoter region of the *IL10* gene has been reported.^(^ ^139^, ^171^ ^)^ Induction of IL‐10 in 1,25D‐treated T‐cell cultures was shown to be increased at lower doses than effects on FOXP3, and in distinct cell populations, suggesting that IL‐10 upregulation is FoxP3 independent.^(^ ^172^ ^)^ IL‐10 induction by 1,25D in CD4^+^ T cells was shown to be partially dependent on a 1,25D‐mediated increase in α‐1‐antitrypsin,^(^ ^173^ ^)^ and α‐1‐antitrypsin has also been shown to induce IL‐10 in DCs,^(^ ^174^ ^)^ suggesting a role for 1,25D‐induced α‐1‐antitrypsin in immunomodulation. The increase in IL‐10 production is then able to indirectly mediate further anti‐inflammatory effects of 1,25D.^(^ ^172^ ^)^ Studies have shown the combination of 1,25D and TGF‐β on CD4^+^ T cells to increase the induction of Tregs above 1,25D used alone, which mechanistically may relate to maintenance of the expression of IL‐2 that is inhibited by 1,25D.^(^ ^139^, ^169^ ^)^ Moreover, it has been shown in vitro that adding IL‐2 to 1,25D‐treated CD4^+^ T cells is able to increase the production of Tregs.^(^ ^169^, ^175^ ^)^

Direct effects of 1,25D on Treg function are less clear, especially considering the contradictory evidence supporting VDR expression in Tregs.^(^ ^124^, ^128^, ^129^ ^)^ Consistent with the low VDR levels described in one study, they also observed no effect of 1,25D on Tregs.^(^ ^129^ ^)^ However in another study, where equal levels of VDR in human T‐regulatory (CD4^+^ CD25^high^) and T‐conventional cells were described, a slight increase in IL‐10 from the Treg population was also observed alongside a decrease in proliferation, suggesting a level of response to 1,25D.^(^ ^128^ ^)^ However, as mentioned previously, it may be that CD4^+^ CD25^high^ is not a reliable identifier of human Tregs. Nevertheless, one study was able to correlate the levels of serum 25(OH)D_3_ with Treg function in patients with MS.^(^ ^176^ ^)^

## Effects of 1,25D on T‐Cell Metabolism

Upon activation, T cells require significant metabolic reprogramming to support their new energetic demands.^(^ ^177^ ^)^ Naïve and memory T cells maintain a catabolic metabolism, supported by oxidative phosphorylation (OXPHOS). In contrast, effector T cells increase nutrient uptake from the environment and levels of glycolysis to switch to an anabolic metabolism that favors biosynthesis of effector molecules and growth.^(^ ^177^, ^178^ ^)^ Changes in metabolism can also directly affect the function of T cells. For example, GAPDH is an enzyme used in glycolysis, but also acts as a transcriptional repressor of *IFNG*. With increased glycolysis, GAPDH is released from binding to *IFNG*, resulting in increased IFN‐γ expression.^(^ ^179^ ^)^ This tight link between cellular metabolism and function also means that each T‐cell subset has a unique metabolic profile, dependent on tissue environment and disease.^(^ ^180^, ^181^ ^)^ T‐cell metabolism can be altered by multiple stimuli, including cytokines, TCR signaling, and cellular stress.^(^ ^178^, ^182^, ^183^, ^184^ ^)^ The impact of 1,25D on T‐cell metabolism has not been explored, but may warrant investigation given substantial effects on metabolism described in other cell types as detailed below.

### Cellular metabolism

In macrophages, 1,25D alters cholesterol metabolism to limit foam‐cell formation in type 2 DM.^(^ ^185^, ^186^ ^)^ Dendritic cells differentiated in the presence of 1,25D form a tolerogenic phenotype (TolDC) with a unique metabolic profile, including induction of pathways of glycolysis and oxidative phosphorylation, forming a highly glycolytic TolDC dependent on the PI3K/Akt/mTOR pathway.^(^ ^187^, ^188^ ^)^ In contrast to upregulation of mTOR, 1,25D caused direct transcriptional upregulation of mTOR inhibitor DDIT4 in osteoblasts, leading to decreased proliferation.^(^ ^189^ ^)^ It is also well‐described that vitamin D increases OXPHOS and maintains mitochondrial function in skeletal muscle.^(^ ^190^, ^191^ ^)^ Most studies looking at the impact of 1,25D on cellular metabolism are in cancer models. Vitamin D is suppressive of glycolysis in multiple cancers.^(^ ^192^, ^193^, ^194^, ^195^ ^)^ For highly glycolytic cancer cells, this inhibition of glucose metabolism is detrimental, resulting in loss of proliferation and increased apoptosis.^(^ ^193^, ^195^, ^196^ ^)^ In several studies, this decrease in glycolysis is linked to inhibition of c‐Myc, a regulator of glycolytic gene transcription, by 1,25D.^(^ ^192^, ^195^, ^196^ ^)^ It has also been reported that 1,25D is able to suppress glutamine metabolism in breast cancer cells, partially through inhibiting transcription of the glutamine transporter.^(^ ^197^ ^)^ In skin, vitamin D can also protect against cancer by increasing glycolysis, autophagy, and mitophagy after UV‐mediated DNA damage to maintain DNA repair in keratinocytes.^(^ ^198^ ^)^

### T‐cell metabolism

Few studies have investigated a role for 1,25D in modulating T‐cell metabolism. In bulk PBMCs isolated from adults with different serum levels of 25D, PMBCs from those in the low vitamin D group had consistently higher basal OXPHOS and basal glycolysis compared with those with higher serum 25D levels.^(^ ^199^ ^)^ A similar study also correlated lower 25D levels in the winter with higher basal PBMC metabolism and increased inflammatory cytokines.^(^ ^200^ ^)^ Although only a correlation—and confounded by multiple additional factors—these data warrant further study into the mechanisms of 1,25D‐mediated metabolic regulation of immune cells. In a study on isolated CD4^+^ T cells, 1,25D treatment for 24 hours caused a reduction in c‐Myc expression.^(^ ^201^ ^)^ This finding had been previously reported in a study that found 1,25D‐mediated inhibition of c‐Myc after 72 hours, but only in the presence of sufficient TCR stimulation to upregulate VDR expression.^(^ ^202^ ^)^ Although not investigated by either study, this decrease in c‐Myc may suppress glycolysis in CD4^+^ T cells.

Transcriptomic analysis of CD4^+^ T cells from EAE mice treated with vitamin D revealed the downregulation of multiple metabolic pathways, including multiple genes from the tricarboxylic acid (TCA) cycle and glycolysis, as well as the PI3K/Akt/mTOR pathway.^(^ ^144^ ^)^ In line with this, another study using CD4^+^ T cells from EAE mice, showed that those treated in vitro with 1,25D had reduced mTOR activity; however, these data were only from a limited number of biological replicates.^(^ ^203^ ^)^ The data also suggested that 1,25D was able to upregulate glucocorticoid‐induced apoptosis, and that in EAE mice with a specific mTOR defect in T cells, this effect was ablated.^(^ ^203^ ^)^ Although a full mechanism was not explored, this study does highlight the need for further studies investigating the effect of 1,25D on T‐cell metabolism in autoimmune inflammatory diseases. Another study of EAE showed that 1,25D increased transcription of BHMT1, an enzyme involved in the conversion of homocysteine to methionine. This upregulation of methionine increased the global DNA methylation status of antigen‐specific CD4^+^ T cells and increased Treg numbers.^(^ ^204^ ^)^

Collectively, these studies show a potential role for 1,25D as a regulator of T‐cell metabolism. The data suggest that 1,25D suppresses T‐cell glycolysis. If confirmed, this reduction may link to the inhibition of IFN‐γ by 1,25D, as glycolysis is known to regulate IFN‐γ expression.^(^ ^179^ ^)^ Although shown to be transcriptionally regulated by vitamin D,^(^ ^154^ ^)^ in some studies little change in *IFNG* mRNA is seen, despite a reduction in cytokine production.^(^ ^150^ ^)^ This suggests additional posttranslational control that may come into effect at different time points or doses.

## Indirect Effects of Vitamin D on T Cells

For T cells, the main source of 1,25D is locally synthesized by APCs expressing the enzyme CYP27B1 and conversion of 25D to 1,25D.^(^ ^146^ ^)^ It has been reported that murine CD8^+^ T cells, but not CD4^+^ T cells, have functional CYP27B1 activity.^(^ ^205^ ^)^ Human CD4^+^ T cells do express functional CYP27B1, although at comparably low levels to dendritic cells.^(^ ^146^, ^206^ ^)^ Vitamin D binding protein (DBP) present in serum also appears to blunt 25D conversion in T cells.^(^ ^146^, ^206^ ^)^ The relevance of CYP27B1 expression by T cells in vivo is not clear. However, it is suggested that the activity level seen in vitro is too low to support any clear changes in cell phenotype.^(^ ^146^ ^)^ Therefore, T cells rely heavily on the local production of 1,25D to reach a sufficient concentration for immunomodulatory effects.^(^ ^146^, ^207^ ^)^ As discussed above, vitamin D exerts a range of effects on innate immune cells. These can then indirectly impact T‐cell differentiation and function.

Treatment with 1,25D has been shown to decrease CD40L expression on macrophages that may limit T‐cell activation and induction of a proinflammatory phenotype.^(^ ^208^ ^)^ Although macrophages are capable of antigen presentation to T cells, studies suggest that dendritic cells are much more influential in this role. During dendritic cell differentiation, treatment with 1,25D leads to development of a tolerogenic phenotype.^(^ ^188^, ^209^ ^)^ TolDCs are less mature than mature dendritic cells and retain the monocyte marker CD14, as well as exhibiting lower expression of costimulatory molecules such as CD80/86 and MHCII/HLA‐DR.^(^ ^188^, ^209^ ^)^ TolDCs have the ability to suppress inflammatory T‐cell proliferation and favor Treg differentiation to restore Th17/Treg balance.^(^ ^207^, ^210^, ^211^ ^)^

TolDCs promote Treg generation through increased IL‐10 production and a lower expression of costimulatory molecules, also preventing antigenic T‐cell activation.^(^ ^14^, ^209^ ^)^ One study has suggested that the ability of 1,25D to increase PFKFB4, an enzyme in glycolysis, is essential for the ability of dendritic cells to induce Tregs.^(^ ^207^ ^)^ This highlights the relevance of 1,25D‐mediated alterations in metabolism in modulating inflammatory cell function in disease. It was reported that 1,25D‐induced TolDCs expressed higher levels of membrane‐bound TNF (mTNF), which is essential for the generation of Tregs.^(^ ^212^ ^)^ Another potential mechanism for 1,25D‐specific induction of TolDCs, is the upregulation of PD‐L1, not seen in dexamethasone‐treated TolDCs, but required for Treg production.^(^ ^210^ ^)^ Overall, it is currently not fully understood how 1,25D‐treated DCs are able to induce high levels of Tregs. Of particular interest is the unique metabolic profile, directly induced by 1,25D,^(^ ^187^, ^188^ ^)^ to TolDC function. It has been reported that in mouse models of autoimmune disease TolDCs are immunosuppressive.^(^ ^14^, ^211^, ^213^ ^)^ Multiple clinical trials have also demonstrated the safety and therapeutic potential for patient administration of TolDC, although often these TolDCs are generated by dexamethasone.^(^ ^214^, ^215^, ^216^ ^)^ Further understanding the mechanisms for 1,25D–TolDCs will be important to move to human trials and develop therapeutic options for 1,25D–TolDCs in inflammatory disease.

## Anti‐Inflammatory Effects of Vitamin D and Autoimmune Disease

Pathogenesis in autoimmunity can be simplified as an increase in proinflammatory factors, often cytokines produced by inflammatory T cells, and a decrease in anti‐inflammatory factors, such as Tregs and IL‐10. Using vitamin D as a therapeutic option for autoimmune inflammatory disease is therefore a promising avenue based on the immunomodulatory actions described earlier in this review, although translating these potential therapies to human use has shown only limited success so far.

### Multiple sclerosis

Because of the well‐described correlation between sunlight exposure and incidence of MS,^(^ ^217^, ^218^ ^)^ there have been a variety of studies on the effects of 1,25D in MS disease models such as EAE. Treatment with 1,25D has been shown to improve EAE symptoms with increases in Th2 cells^(^ ^150^ ^)^ and decreases in Th1 and Th17 cells.^(^ ^144^, ^156^, ^175^ ^)^ Studies have also confirmed the essential role of VDR expression in CD4^+^ T cells, and the expression of IL‐10 to mediate the protective effects of 1,25D in EAE.^(^ ^219^, ^220^ ^)^ A potential synergy of 1,25D and estrogen has been suggested by one EAE study, causing an increase in Tregs.^(^ ^221^ ^)^ This may be an important factor in the sex bias of MS incidence and for potential stratification of patient treatment.^(^ ^221^ ^)^ In patients with MS, it has been reported that the ratio of serum 1,25D:25D, but not 1,25D or 25D alone, correlates directly with peripheral Treg percentages.^(^ ^176^, ^222^ ^)^ It was also demonstrated that vitamin D treatment increased Treg numbers in healthy adults.^(^ ^223^ ^)^ One study showed correlation of cytokine levels to disease progression, showing lower levels of inflammatory cytokines, such as IFN‐γ and IL‐17, associated with better disease scores.^(^ ^164^ ^)^ In this case, 1,25D treatment of CD4^+^ T cells from patients with MS in vitro was able to reduce the production of inflammatory cytokines.^(^ ^164^ ^)^ In clinical trials, the effects of vitamin D have been more modest. A trial of patients with relapsing–remitting MS (RRMS) treated with vitamin D_3_ (cholecalciferol) showed no improvement, but suggested that there may be a benefit in patients already treated with IFN‐β.^(^ ^224^ ^)^ However, in a trial involving patients with RRMS on IFN‐β therapy, no difference was observed in proportions of T‐cell subsets with vitamin D_3_ treatment.^(^ ^225^ ^)^ Moreover, in another trial on RRMS patients treated with IFN‐β, no additional effect on patient recovery was seen upon vitamin D_3_ treatment, but it was suggested that there was a potential improvement observed on development of new lesions.^(^ ^226^ ^)^

### Rheumatoid arthritis

In a mouse model of RA, collagen‐induced arthritis (CIA), 1,25D treatment showed significant effects, completely halting the progression of the disease.^(^ ^227^ ^)^ This improvement was linked to changes in multiple cell types, including synovial fibroblasts and monocytes, and likely involved a range of 1,25D‐mediated immunomodulatory effects.^(^ ^228^, ^229^, ^230^ ^)^ Another study using CIA mice showed 1,25D treatment to decrease the production of IL‐17 and increase regulatory cells in the synovial fluid of diseased mice.^(^ ^231^ ^)^ This study suggested a novel mechanism of microRNA induction by 1,25D, which then inhibited IL‐6 signaling, a key inflammatory cytokine in RA.^(^ ^231^ ^)^

Other studies have focused on in vitro treatment of human RA CD4^+^ T cells, where RA Th17 cells are able to differentiate into regulatory cells when cultured with 1,25D.^(^ ^158^, ^162^ ^)^ These memory Th17 cells, although losing CCR6 expression, were still able to migrate to inflamed synovial fluid, showing promise for future therapeutic use.^(^ ^162^ ^)^ Additionally, RA T‐cell‐synovial fibroblast cocultures have shown that, in combination with TNF‐α blockade, a 1,25D‐mediated decrease in IL‐17 aids in dampening synovial inflammation.^(^ ^232^ ^)^ Although multiple in vitro effects have been reported for 1,25D and RA, more trials and human studies evaluating a role for vitamin D supplementation in treating inflammatory RA are required. This is highlighted in a recent study, which showed that CD4^+^ T cells from RA synovial fluid are less sensitive to suppression by 1,25D than those from peripheral blood,^(^ ^233^ ^)^ an important factor to consider when developing vitamin D therapy.

### Systemic lupus erythematosus

T cells are instrumental in SLE pathogenesis, aiding B‐cell responses and amplifying inflammation. Because of the systemic nature of SLE, in vitro and animal studies are more limited.^(^ ^234^, ^235^, ^236^ ^)^ However, vitamin D_3_ therapy has shown considerable success in trials with patients with SLE. Clinical studies focused on disease activity and fatigue in patients with SLE have found that vitamin D_3_ supplementation has beneficial effects.^(^ ^237^ ^)^ Several of these trials have also reported alterations to T cells after vitamin D treatment. One relatively large study of 267 patients showed a significant decrease in inflammatory cytokines, IL‐18, IL‐1, IL‐6, and TNF‐α, in the vitamin‐D‐treatment group.^(^ ^238^ ^)^ Multiple studies have also identified an increase in Tregs with vitamin D treatment, with less‐consistent decreases in Th17 cells and Th1 cells and increases in Th2 cells.^(^ ^239^, ^240^, ^241^ ^)^ In contrast, a study evaluating the effect of vitamin D on the IFN signature in patients, a key marker of SLE pathogenesis, showed no differences between vitamin D and placebo groups.^(^ ^242^ ^)^ However, this was a relatively short‐term study of 12 weeks and analyzed the expression of only three signature genes.^(^ ^242^ ^)^

### Inflammatory bowel disease

Mouse models of IBD have identified multiple anti‐inflammatory effects of vitamin D in the gut.^(^ ^243^, ^244^, ^245^ ^)^ A study, using the trinitrobenzene sulfonic acid (TNBS) IBD model with vitamin D treatment, showed increases in IL‐10, IL‐4, TGF‐β, and IL‐4 along with a decrease in Th1 cells.^(^ ^149^ ^)^ Another common model of IBD is the use of IL‐10 KO mice. In these studies using these mice, although lacking the increase in IL‐10 associated with 1,25D activity, vitamin D supplementation was nonetheless able to improve symptoms and decrease TNF‐α expression.^(^ ^244^, ^245^ ^)^ Patients with Crohn disease exhibited similar results to the mouse studies with increased IL‐10 and decreased IFN‐γ production upon 1,25D treatment.^(^ ^246^ ^)^ Vitamin D supplementation trials for IBD have shown some benefit in suppressing inflammatory disease score.^(^ ^247^, ^248^, ^249^, ^250^ ^)^ However, additional parameters such as cytokine levels have yet to be investigated. Of particular interest is the potential use of vitamin D as a supplement to anti‐TNF‐α treatment to improve immunomodulation in patients with IBD.^(^ ^251^, ^252^ ^)^ Currently, trials have focused on and reported a reduction in IBD disease progression with the view to move to larger cohort studies.^(^ ^248^, ^249^ ^)^

### Type 1 diabetes mellitus

Consistent with the other mouse models of chronic autoimmunity, vitamin D treatment of nonobese diabetic (NOD) mice has been shown to alleviate DM by reducing the production of IFN‐γ, increasing Treg numbers, and restoring defective T‐cell apoptosis.^(^ ^148^, ^211^, ^253^ ^)^ In contrast, a study using VDR^−/−^ NOD mice showed that mice lacking the VDR were no worse off in DM presentation, suggesting that vitamin D has little effect in disease progression.^(^ ^254^ ^)^ Human studies are similarly contradictory, two studies have reported no association with pregnancy/neonatal 25D levels and risk of developing T1DM,^(^ ^255^, ^256^ ^)^ whereas another larger study reported a reduced risk of developing T1DM with neonatal vitamin D supplementation.^(^ ^257^ ^)^ Several adult cohort studies have investigated a potential protective effect of vitamin D on β‐cell function. Two studies showed that 1,25D provided no protection to β cells,^258^, ^259^ ^)^ but another suggested β‐cell function is preserved with a combination of insulin and 1α‐hydroxyvitamin D_3_ therapy.^(^ ^260^ ^)^ In the only study to report changes in the T‐cell compartment, an increase in Tregs was reported in males treated with vitamin D_3._^(^ ^261^ ^)^ This provides a good rationale for further studies investigating vitamin D‐mediated improvements in T‐cell function in DM and highlights a potential sex bias in some effects.^(^ ^261^, ^262^ ^)^

### Airway disease

In several studies, lower circulating 25D levels have been reported in individuals with severe asthma.^(^ ^263^ ^)^ Moreover, in one study, patients with the lowest baseline 25D levels were found to be at highest risk of severe asthma exacerbation requiring hospital admission over a 4‐year period, which may indicate some degree of causality.^(^ ^264^ ^)^ Studies aimed at identifying a mechanistic basis for this relationship have identified positive correlations between vitamin D status and both the frequency of FoxP3^+^ regulatory T cells and levels of the anti‐inflammatory cytokine IL‐10 in the airways of patients with asthma,^(^ ^172^, ^265^ ^)^ consistent with the capacity of 1,25D to drive differentiation of CD4^+^ T cell populations in vitro.^(^ ^126^, ^172^ ^)^ Effects on other types of cells present in the airway may also partly explain these relationships: For example, 1,25D suppresses production of IgE by B lymphocytes^(266)^ and of inflammatory cytokines by neutrophils,^(76^ ^)^ but promotes epithelial cell secretion of anti‐inflammatory ST2, which blocks mast cell priming by IL‐33.^(^ ^267^ ^)^ Notably, 1,25D also augments the sensitivity of both T cells and monocytes to the anti‐inflammatory activity of corticosteroids, which are a mainstay of treatment of this disease.^(^ ^268^, ^269^, ^270^ ^)^ Despite these in vitro findings and the relationship between vitamin D status and asthma severity described above, interventional randomized controlled trials of vitamin D supplementation in respiratory disease have so far produced inconsistent results in adults, although more encouraging results have been described in children.^(^ ^263^ ^)^

Although studies of the effects of vitamin D on inflammatory disease using mouse models have provided mechanistic insight into the immune functions of 1,25D, and support the further investigation of possible benefits of vitamin D supplementation in human inflammatory disease, there are several caveats to consider. First, as detailed earlier, it is clear that some immune responses to vitamin D, in particular innate immune responses, may show significant differences between mice and humans.^(^ ^45^ ^)^ It is also important to recognize that although murine models of immune disease have focused on the effects of enhanced 1,25D or VDR knockout on inflammation, most human studies have centered on the effects of vitamin D deficiency (low serum 25D) or vitamin D supplementation and associated increases in serum 25D. Direct therapeutic administration of 1,25D in humans is complicated by well‐established hypercalcemic side‐effects. Therefore, human studies have relied on the fundamental assumption that vitamin D supplementation, leading to increased serum 25D, will also result in increased localized synthesis of 1,25D commensurate with direct administration of 1,25D as commonly used in mice. Conversely, studies of vitamin D deficiency in humans are assumed to mimic some elements of VDR ablation in mice. Despite this dichotomy in experimental strategies for vitamin D in humans and mice, there have been some studies of the effects of vitamin D deficiency on mouse immune function that parallel human studies. Dietary vitamin D deficiency and low serum 25D in mice have been shown to result in dysregulated inflammation in mouse models of IBD,^(^ ^271^ ^)^ similar to the effects of VDR knockout. However, in other autoimmune disorders such as MS, the EAE mouse model showed variable responses to vitamin D deficiency, with reports describing both protective effects of vitamin D deficiency^(^ ^272^ ^)^ and increased disease severity,^(^ ^273^ ^)^ depending on when the deficiency occurred relative to disease induction. These observations highlight an important objective for future studies of vitamin D, namely the development of animal models that better reflect the likely application of vitamin D in either the prevention or treatment of immune diseases in humans.

## Vitamin D and Antiviral Innate Immunity

Mechanisms of antiviral innate immunity parallel those that counter bacterial infections; viral pathogens are first detected through a series of pattern recognition receptors, which initiate signaling cascades that induce type 1 interferon and cytokine responses, as well as antiviral effectors.^(^ ^274^, ^275^ ^)^ Several endosomal or intracellular PRRs are activated by various forms of nucleic acids, such as double‐stranded RNA (dsRNA), and act as sensors of invading viral genomes. Initial transcriptional responses to detection of viral pathogens are channeled through a limited number of transcription factors, notably, members of the interferon regulatory factor family such as IRF3 and IRF7, AP‐1 family members, and NF‐kB.^(^ ^276^, ^277^ ^)^ The initial transcriptional wave and downstream interferon signaling give rise to the production of a number of antiviral effectors.

Numerous epidemiological and clinical studies have examined links between vitamin D status and immune protection against several classes of viruses, notably several viral respiratory pathogens, hepatitis viruses, human immunodeficiency virus, and herpes simplex viruses. For a recent comprehensive overview of clinical evidence for vitamin D signaling in antiviral immunity, readers are referred to the review by Lee.^(^ ^278^ ^)^ We will focus here on vitamin D signaling in viral respiratory tract infections. Available epidemiological data support a role for vitamin D in reducing rates of acute respiratory tract infections. A meta‐analysis by Martineau and colleagues found that daily or weekly vitamin D supplementation reduced the incidence of such infections, and that the effects were most pronounced in subjects with the lowest levels of circulating 25D.^(^ ^279^ ^)^ One of the most compelling studies in this regard was a randomized placebo‐controlled trial in Japanese school children, wherein supplementation diminished the incidence of seasonal influenza A (InA), with the most striking results in a subgroup that had not received supplements prior to the trial.^(^ ^280^ ^)^

Many respiratory viruses target lung epithelial cells, which are highly vitamin D responsive. The bulk of the evidence suggests that vitamin D signaling boosts primary antiviral immunity, while suppressing proinflammatory cytokine responses. Hansdottir and colleagues^(^ ^281^ ^)^ found that primary human lung epithelial cells express CYP27B1, and that, importantly, its expression was enhanced by dsRNA. 1,25D strongly induced expression of *CAMP* and *CD14*, and evidence was presented that proinflammatory NF‐κB signaling was downregulated. Similarly, Telcian and colleagues found that respiratory syncytial virus (RSV) infection of bronchial epithelial cells induced 1α‐hydroxylase activity.^(^ ^282^ ^)^ One of the key elements of 1,25D‐mediated antiviral responses is the robust induction of *CAMP* expression. The secreted form of CAMP, LL‐37, appears to exert antiviral activity by multiple mechanisms. For example, LL‐37 augments signaling the PRR TLR3, a detector of viral dsRNA. Lai and colleagues found that LL‐37 stimulated TLR3 signaling induced by poly(I:C) and facilitated recognition of viral dsRNAs by TLR3.^(^ ^283^ ^)^ Subsequent work provided evidence that LL‐37, an amphipathic cationic peptide, bound directly to dsRNA and trafficked to endosomes where TLR3 is located. Dissociation of LL‐37‐bound dsRNA occurred in acidic endosomal compartments.^(^ ^284^ ^)^

LL‐37 may also have direct antiviral activity against InA virus. Tripathi and colleagues found that LL‐37 binds directly to InA viruses, and electron microscopy data suggested that LL‐37 directly disrupted viral membranes.^(^ ^285^ ^)^ Similar evidence was found for a direct antiviral effect of human and murine forms of CAMP in mouse models of InA infection. Moreover, treatment of infected mice with LL‐37 reduced pulmonary levels of proinflammatory cytokines.^(^ ^286^ ^)^ Likewise, LL‐37 has antiviral activity against RSV in vitro. Treatment of infected epithelial cells prevented virus‐induced cell death and suppressed viral replication via inhibition of assembly of viral particles.^(^ ^287^ ^)^ There may also be a role for another 1,25D‐inducible AMP, HBD2/DEFB4, in antiviral responses. HBD2 expression is induced by RSV infection in lung epithelial cells, and in vitro studies showed that it had direct antiviral activity by blocking viral entry possibly via disruption of the integrity of the viral envelope.^(^ ^288^ ^)^ In another study by Hansdottir and colleagues,^(^ ^289^ ^)^ vitamin D signaling enhanced production of NF‐κB inhibitor IKBα and suppressed expression of NF‐κB target genes in RSV‐infected cells. The effect of 1,25D could be mimicked by transduction of a nondegradable form of IKBα. Importantly, treatment with 1,25D did not lead to an increased viral load, leading to the conclusion that vitamin D suppressed inflammatory responses without compromising host defense in infected cells.^(^ ^289^ ^)^ Similar conclusions were reached by Stoppelenburg and colleagues in a model of RSV infection in which the VDR was expressed in A549 human lung epithelial cells.^(^ ^290^ ^)^ Available results for rhinovirus infection are mixed. In one study using primary human bronchial epithelial cells, no significant effect of 1,25D on rhinovirus viability was observed.^(^ ^291^ ^)^ Conversely, in other experiments in bronchial epithelial cells, rhinovirus infection decreased expression of the VDR and CYP24A1, whereas 1,25D treatment decreased rhinovirus replication and release. The effect of 1,25D was accompanied by increased expression of *CAMP* and interferon‐stimulated genes.^(^ ^282^ ^)^

Other facets of the antibacterial mechanisms that are activated by vitamin D may also play a pivotal role in mediating its antiviral functions. NOD2 is known to recognize single‐stranded RNA to trigger type I interferon antiviral responses.^(^ ^292^ ^)^ Thus, the ability of 1,25D to strongly induce NOD2 expression^(^ ^52^ ^)^ may indirectly enhance antiviral activity. NOD2 is also known to trigger autophagy,^(^ ^57^ ^)^ and autophagy has been shown to be induced by herpes simplex virus and human cytomegalovirus herpes virus infection.^(^ ^293^ ^)^ Thus, the potent effects of 1,25D in promoting autophagy to enhance the intracellular environment for bacterial killing may also play a role in antiviral activity by facilitating viral clearance.^(^ ^294^, ^295^ ^)^ By packaging viral particles for lysosomal degradation and antigen‐presentation, autophagic encapsulation provides an initial step in development of adaptive antiviral immune responses.^(^ ^296^ ^)^ However, it is important to recognize that some viruses are able to hijack components of autophagy to promote viral replication. For example, hepatitis C virus infection is known to promote autophagy,^(^ ^297^ ^)^ but the resulting lipid‐filled autophagosomes can act to enhance the assembly of hepatitis C virions.^(^ ^298^ ^)^

Autophagy is another key mechanism for controlling viral infection and replication, and both 25D and 1,25D have been shown to enhance macrophage expression of the autophagy marker LC3.^(^ ^31^, ^58^ ^)^ Thus, autophagy is subject to the same localized innate immune intracrine vitamin D metabolic system observed for antibacterial proteins.^(^ ^299^ ^)^ Vitamin D can promote autophagy first by direct induction of key enzyme drivers of autophagy such as promoting Beclin 1 and PI3KC3.^(^ ^300^ ^)^ However, it can also enhance autophagy indirectly by suppressing inhibition of autophagy by the mTOR signaling pathway,^(^ ^301^ ^)^ and by stimulating intracellular calcium and nitric oxide (NO) by 1,25D to enhance PI3KC3 activity.^(^ ^302^ ^)^ Another key indirect mechanism for 1,25D‐mediated induction of autophagy is via cathelicidin expression, which, in turn, stimulates key autophagy factors such as Beclin 1.^(^ ^58^ ^)^ The overall autophagy effect of vitamin D appears to be as a homeostatic regulator of the balance between autophagy and apoptosis to enable optimal antiviral responses to infection.^(^ ^303^ ^)^ The potential benefit of vitamin‐D‐induced autophagy as an inhibitory mechanism to counter viral infection has been reviewed in detail elsewhere,^(^ ^303^ ^)^ and notably includes beneficial effects on infection by hepatitis C,^(^ ^304^ ^)^ InA,^(^ ^305^ ^)^ rotavirus,^(^ ^306^ ^)^ and HIV‐1.^(^ ^307^, ^308^ ^)^

Comorbidities, such as COPD and asthma, can aggravate respiratory infections, and available, albeit partially conflicting, results suggest that vitamin D metabolism is disrupted in these conditions. TGF‐β1 levels are elevated in COPD and suppress expression of host defense mediators. Schrumpf and colleagues investigated the combined effects of 1,25D and TGF‐β1 on airway epithelial cell host defenses in vitro.^(^ ^309^ ^)^ TGF‐β1 inhibited 1,25D‐induced *CAMP* expression in part by augmentation of CYP24A1 production. TGF‐β1 also attenuated the expression of vitamin D‐independent defense mediators, consistent with an attenuation of vitamin D‐dependent and vitamin D‐independent host defenses.^(^ ^309^ ^)^ However, these findings were not borne out by the results of a longitudinal study by Jolliffe and colleagues,^(^ ^310^ ^)^ who found vitamin D supplementation did not augment circulating 25D levels to the same degree in patients with COPD or asthma as in healthy controls. This may have been because of reduced 25‐hydroxylation of vitamin D, but could not be attributed to elevated 25D catabolism in COPD, as CYP24A1 levels were somewhat reduced in patients with COPD. 1,25D‐dependent gene expression signatures were also modestly attenuated in COPD.^(^ ^310^ ^)^

## Vitamin D and COVID‐19

Given the extensive number of reports establishing an antiviral role for vitamin D signaling, it is reasonable to speculate the vitamin D sufficiency may have a role in attenuating the current outbreak of the novel severe acute respiratory syndrome coronavirus 2 (SARS‐CoV‐2) that is causing the widespread COVID‐19. Although randomized controlled trials and large population studies assessing vitamin D status and severity of COVID‐19 have yet to be completed, there is existing evidence to suggest that vitamin D could exert a protective effect against the disease. SARS‐CoV‐2 initially uses immune evasion mechanisms, which in certain patients, is accompanied with elevated proinflammatory cytokine release, increased risk of pneumonia,^(^ ^311^ ^)^ sepsis, and subsequent acute respiratory distress syndrome (ARDS) that frequently results in mortality.^(^ ^312^ ^)^ In this regard, there is evidence for a protective role of vitamin D in many conditions associated with pneumonia, cytokine hyperproduction, and ARDS.^(^ ^313^, ^314^ ^)^ 1,25D has also recently been proposed as a repurposed drug for InA H5N1 virus‐induced lung injury, an infectious disease with characteristic features similar to COVID‐19.^(^ ^315^ ^)^ The study found that vitamin D supplementation in H5N1 virus‐infected mice decreased the lung injury score, improved mouse lung edema, and increased survival of H5N1‐virus–infected mice. Moreover, vitamin D treatment was beneficial in animal models of ARDS as 1,25D was shown to reduce LPS‐induced–increased lung permeability by regulating activity of the renin‐angiotensin system and expression of angiotensin converting enzyme 2 (ACE2), the host cell receptor responsible for mediating infection by SARS‐CoV‐2.^(^ ^316^ ^)^ Consistent with this, gene‐set–enrichment analysis of genomic data sets identified the VDR as being coexpressed with both *ACE2* and *FURIN* genes, both of which have a key role in promoting high‐affinity binding of viruses and their entry into human cells, in human tissues.^(^ ^317^ ^)^ The study also reported that 1,25D altered expression of genes encoding human proteins for 19 of 27 (70%) of SARS‐CoV‐2 proteins.

Vitamin D deficiency in individuals self‐isolating because of the COVID‐19 pandemic may be aggravated by confinement indoors, a condition that could be reversed by short, daily exposures to the sun in the middle of day.^(^ ^318^ ^)^ Moderate exposure to sunlight would promote sufficient vitamin D production in the skin from March through to September in most countries in northern latitudes. Several epidemiological findings support a therapeutic role for vitamin D in COVID‐19 pathogenesis. To illustrate, the number of cases in countries in the southern hemisphere, specifically those that lie below 35° North, have a relatively lower mortality rate.^(^ ^319^ ^)^ As an example, Australia is 1 week behind the United Kingdom in the spread of the virus; however, if one compares the mortality (68 per million) in the United Kingdom with the mortality (2 per million) in Australia at corresponding dates in the pandemic, there is a large discrepancy. It is important to note that there are outliers; for instance, mortality is low in Scandinavian countries that instituted confinement protocols such as Norway. However, the country is relatively thinly populated and vitamin D insufficiency is rare because of widespread consumption of cod liver oil supplements, as well as fortification of milk and dairy products.^(^ ^320^ ^)^ Conversely, Italy and Spain have higher total death counts, as well as a relatively high prevalence of vitamin D deficiency, possibly because of the population's strong preference of shade under the strong sun,^(321^ ^)^ as well as darker skin pigmentation, which decreases vitamin D synthesis.^(^ ^322^ ^)^ Another trend observed is the number of COVID‐19 case‐fatality rates that increase with age and chronic disease comorbidity, both of which are associated with lower serum 25D levels.^(^ ^323^, ^324^, ^325^, ^326^ ^)^

One recent study suggested a crude negative correlation between mean vitamin D levels in European countries and the number of COVID‐19 cases and deaths caused by the disease^(^ ^327^ ^)^; in contrast, another group that explored COVID‐19 transmission and UV radiation in 62 Chinese cities did not find evidence for such an association.^(^ ^328^ ^)^ However, as mentioned earlier, randomized controlled trials and large population studies are still required to evaluate whether vitamin D could be of benefit, as well as whether it could be used as a preventive or a therapeutic measure. Given that ethnic minorities are disproportionately affected by COVID‐19 in the United Kingdom, the United States, and other countries in Europe, further research is warranted, especially because there is evidence for vitamin D deficiency in these ethnic groups.^(^ ^329^, ^330^ ^)^ At this writing, there are 18 clinical trials registered (www.clinicaltrials.gov) investigating links between vitamin D and COVID‐19,^(^ ^331^, ^332^, ^333^, ^334^, ^335^, ^336^, ^337^, ^338^, ^339^, ^340^, ^341^, ^342^, ^343^, ^344^ ^)^ a number that is growing rapidly.

## Conclusions

There is strong evidence that vitamin D metabolic enzymes are expressed in virtually all cells in the innate and adaptive arms of the immune system. Considering the findings discussed above, vitamin D signaling appears to influence susceptibility to and severity of bacterial and viral infection via several mechanisms. These include its direct effects on the production of antimicrobial peptides and cytokines, as well as its regulation of the NF‐κB pathway during infection. Overall, preclinical and clinical data propose a strong link between vitamin D status and susceptibility to infectious and autoimmune diseases. There is evidence that vitamin D deficiency during early life may predispose the immune system to a greater risk of autoimmune disease or allergy.^(^ ^345^ ^)^ Several laboratory and clinical studies have provided support for a role of vitamin D in combating respiratory tract infections. Evaluation of vitamin D supplementation as an adjuvant therapeutic intervention could be clinically and economically significant in the ongoing COVID‐19 crisis, as well as in the treatment of other infectious diseases. Based on the immunoregulatory properties of vitamin D presented above, improving circulating 25D levels may slow progression of disease or even ameliorate patient survival. Though the evidence for a mechanistic role for vitamin D signaling in immune system regulation is highly compelling, there is still a need for large‐scale randomized controlled trials to confirm whether maintaining vitamin D sufficiency reduces the incidence and severity of infections and/or autoimmune diseases.

## Disclosures

The authors have no conflicts of interest to declare.

Acknowledgments

## Authors roles

ELB, AI, and SKD were involved in the conceptualization, visualization, writing, reviewing, and editing of the manuscript. MH and JHW were involved in conceptualization, investigation, and administration of the project, as well as the supervision, visualization, writing, review, and editing of the manuscript.

## Author Contributions

**Emma Bishop:** Conceptualization; visualization; writing‐original draft; writing‐review and editing. **Aiten Ismailova:** Conceptualization; visualization; writing‐original draft; writing‐review and editing. **Sarah Dimeloe:** Conceptualization; visualization; writing‐original draft; writing‐review and editing. **Martin Hewison:** Conceptualization; investigation; project administration; supervision; visualization; writing‐original draft; writing‐review and editing. **John White:** Conceptualization; formal analysis; investigation; project administration; resources; supervision; visualization; writing‐original draft; writing‐review and editing.

### Peer Review

The peer review history for this article is available at https://publons.com/publon/10.1002/jbm4.10405.
